# Passive fit and trueness of complete-arch implant-supported cobalt chromium frameworks manufactured using different digital techniques: a laboratory study

**DOI:** 10.1186/s12903-026-08524-y

**Published:** 2026-05-13

**Authors:** Esmaeil Mostafa Ahmed, Yasmine Galaleldin Thabet, Amany Mostafa Saad Farahat

**Affiliations:** Department of Oral and Maxillofacial Prosthodontics, Faculty of Dentistry, Ain Shams University, Organization of African Unity Street, Cairo Governorate, Cairo, Egypt

**Keywords:** Cobalt chromium frameworks, Milling, 3D printing, Passive fit, One-screw test, Trueness, Complete arch implant-supported prosthesis

## Abstract

**Background:**

Passive fit and trueness are keys to the success of complete-arch implant-supported cobalt chromium frameworks, so this study aimed to assess and compare the passive fit and trueness of cobalt chromium (Co-Cr) complete-arch implant-supported frameworks fabricated using milling and three-dimensional (3D) printing technologies with those produced using the conventional lost wax technique.

**Methods:**

A maxillary typodont with 5 implant digital analogs was scanned using a desktop scanner, a complete-arch implant-supported framework was designed, and the design’s standard tessellation language (STL) file was designated as the reference file. A total of 24 frameworks were fabricated. In the first group, the frameworks were initially milled from wax blanks and subsequently processed using the conventional lost wax technique. In the second group, frameworks were milled from Co-Cr blanks, while in the third group, frameworks were 3D printed utilizing the selective laser melting technique from Co-Cr powder. Frameworks were scanned, and corresponding STL files were imported into a surface-matching software program to assess trueness. Passive fit was evaluated using a one-screw test and a handheld digital microscope. Statistical analysis was performed using one-way and two-way ANOVA followed by the Tukey post hoc test (α = 0.05) for pairwise comparison.

**Results:**

Significant differences were found among fabrication techniques for both passive fit and trueness. The milled group demonstrated the lowest mean of vertical gap during passive fit assessment, while the conventional lost wax group exhibited the highest mean. Correspondingly, trueness values were lowest for the milled frameworks, intermediate for 3D-printed frameworks, and highest for conventional lost-wax.

**Conclusions:**

Digital fabrication techniques, particularly CAD-CAM milling, provided superior fit and trueness compared to conventional casting of Co-Cr complete-arch implant frameworks.

**Graphical abstract:**

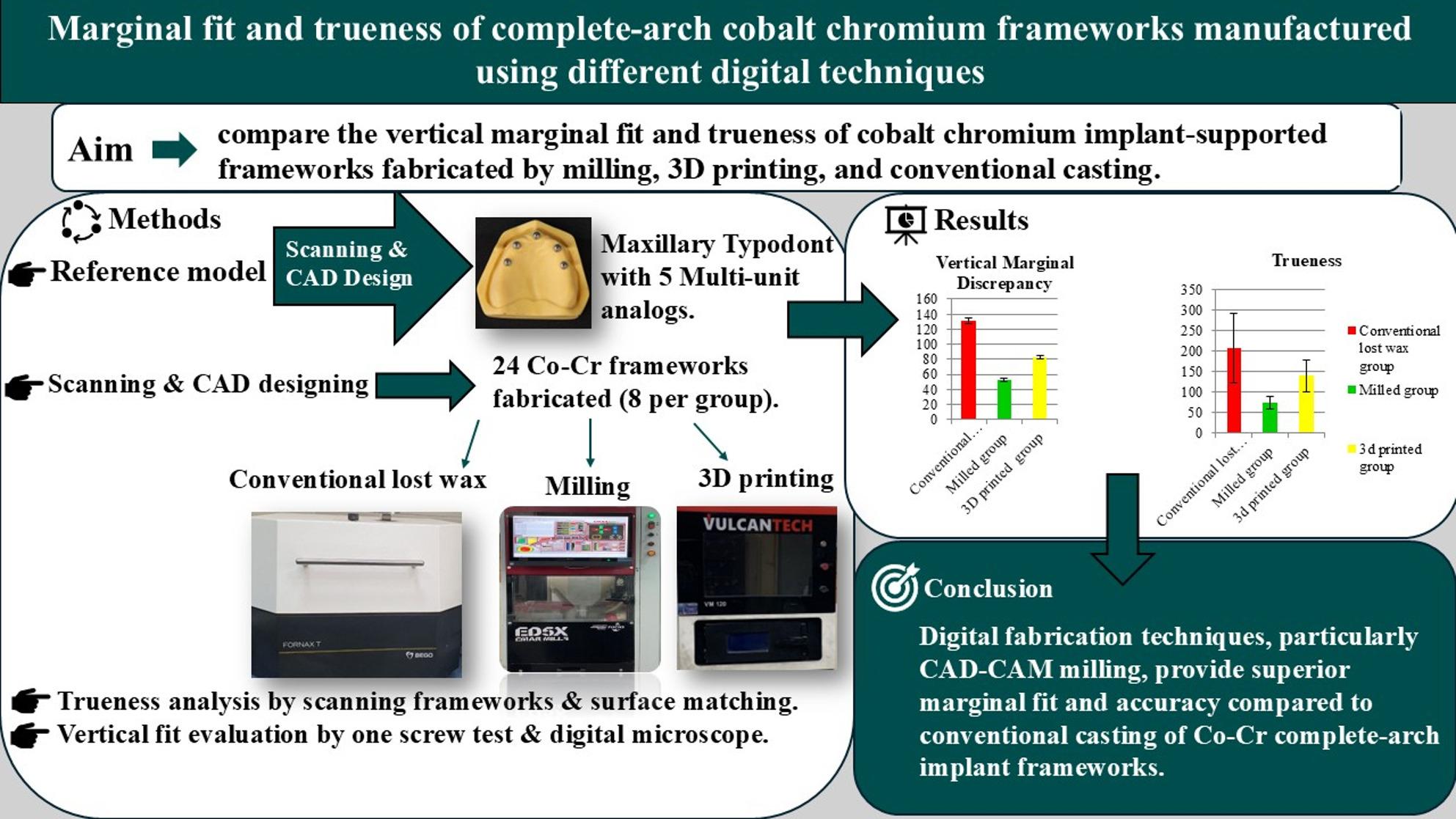

## Highlights

Co-Cr frameworks were fabricated using casting, milling and SLM 3D printing.

Fabrication method significantly affected marginal fir and trueness.

Milled frameworks showed best marginal fit and trueness.

SLM frameworks showed intermediate trueness versus conventional casting.

CAD-CAM milling achieved higher accuracy for complete-arch Co-Cr frameworks.

## Background

Implant prosthetics provide established approaches for complete arch cases; however, successful outcomes rely on precise fabrication of prosthetic frameworks to align with the biomechanical properties of the implants and the jaws into which they are integrated [1]. Cobalt-chromium (Co-Cr) alloys are commonly used for implant frameworks due to their high mechanical properties and economic advantage, although their high hardness may complicate the finishing procedure [2]. Conventional lost wax casting is widely used, but as a multi-step, technique-sensitive process, it introduces multiple variables affecting outcomes [3, 4].

The introduction of computer-aided design and computer-aided manufacturing (CAD-CAM) technology utilizing both subtractive (milling) and additive (3D printing) manufacturing techniques revolutionized the fabrication of Co-Cr restorations [5, 6]. A hybrid workflow was used to minimize the technique-sensitive inaccuracies associated with the conventional lost wax technique, where the framework was digitally designed and milled from wax blanks before being invested and cast [6, 7]. This helps minimize laboratory-related errors and improve the accuracy of the final framework. The milling technique also permits the direct fabrication of Co-Cr frameworks from solid blanks, providing a fully digital workflow with improved adaptation, precision, and fewer laboratory steps compared to lost wax casting [8]. However, material hardness results in rapid deterioration of the milling tools, which, eventually, affects the accuracy of the frameworks. Additionally, its high cost represents another major drawback of this technique [9, 10].

Three-dimensional printing allows the production of complex art beyond subtractive method limitations. Integrated with CAD, selective laser melting (SLM) uses a focused laser to fuse fine metal powder layers, directly producing Co-Cr restorations from digital designs [11]. This technique operates by melting fine layers of metal powder with a concentrated high-power laser beam [12, 13]. However, the high cost of the required equipment currently confines its use to major CAD-CAM laboratories in the dental field [9, 10, 14, 15].

Long-term success of implant-supported prostheses requires both passive fit and structural accuracy [16, 17]. Passive fit is defined as even and simultaneous contact between the inner surface of the framework retainers and the supporting implant superstructure while inducing zero strain on the supporting implants and the surrounding residual bone in the absence of any external load or force [18–20]. Despite all trials, new technologies, materials, and recent fabrication techniques, achieving passive fit as defined previously is not applicable [21, 22]. Lack of passive fit can precipitate mechanical complications, such as screw loosening and prosthetic component fracture, and biological complications, including plaque accumulation, peri-implant mucositis, peri-implantitis, marginal bone loss, and eventual loss of osseointegration [23–25]. These adverse outcomes not only compromise the functional integrity of the restoration but also jeopardize peri-implant tissue health [26–28].

Accuracy, as defined by the International Organization for Standardization ISO 5725-1 [29], encompasses both precision and trueness. Trueness is defined as the degree of agreement between the arithmetic mean of a substantial number of test outcomes and the true or accepted reference value. Precision denotes the degree of agreement among independent test results obtained under specified conditions; it serves as a broad term encompassing the variability observed in repeated measurements. In the present study, trueness was selected to be evaluated, as it provides a quantitative 3D assessment of how closely the definitive prosthesis reproduces the reference design, allowing standardized comparison among different manufacturing techniques. Precision was not assessed, as the objective was to evaluate deviation from the intended geometry rather than the consistency of the repeated fabrications.

Although different manufacturing techniques have been investigated for Co-Cr implant-supported frameworks, most of these studies have focused on single outcome measures, such as marginal or internal adaptation, or have been limited to short-span prostheses [27, 30]. Such conditions do not represent the clinical complexity of complete-arch frameworks, where dimensional discrepancies tend to accumulate over extended spans. To the best of the authors’ knowledge, no previous study has concurrently evaluated both passive fit and trueness in complete-arch Co-Cr implant-supported frameworks fabricated using different techniques. Therefore, the present study was designed to assess and compare the passive fit and trueness of complete-arch implant-supported Co-Cr frameworks fabricated using different manufacturing techniques. Accordingly, two null hypotheses were formulated. The first null hypothesis stated that the manufacturing techniques had no statistically significant effect on the passive fit of Co-Cr frameworks among the tested groups. The second null hypothesis stated that the manufacturing techniques had no statistically significant effect on the trueness of Co-Cr frameworks.

## Methods

### Study design and sample size

This laboratory study was carried out on a resin 3D-printed edentulous maxillary typodont with 5 equidistant parallel straight multi-unit implant analogs. In the first group, the frameworks were initially milled from wax blanks and subsequently processed using the conventional lost wax technique. In the second group, frameworks were milled from Co-Cr blanks, while in the third group, frameworks were 3D-printed utilizing the SLM technique from Co-Cr powder. Table 1. presents a comprehensive overview of the materials used, their compositions, and processing methods. Power analysis was designed to have adequate power to apply a statistical test of the null hypothesis that there is no difference between tested groups regarding passive fit. The analysis was performed using the G*Power software program (version 3.1.9.7; Heinrich-Heine University, Düsseldorf, Germany) to determine the sample size. Based on α = 0.05, β = 0.2, and an effect size (0.682) obtained from a previous study [6], a total of 24 specimens (*n* = 8) was determined to be adequate. Passive fit was considered the primary outcome variable for sample size estimation. Although multiple variables were assessed, these were treated as secondary outcomes.

**Table 1:** A comprehensive overview of used materials, their compositions, and processing methods

Scientific name	Incinerable acrylate polymer blanks	Dental cobalt-based metal-ceramic alloy Type 4	Cobalt-chromium CAD-CAM milling blanks	Cobalt-chromium dental alloy powder
Brand name	IPS Acryl CAD Blocks	Wirobond 280	Scheftner Starbond Easy Disc	Starbond Easy Powder 30
Manufacturer	Ivoclar Vivadent	BEGO	Scheftner GmbH (S&S Scheftner)	Scheftner GmbH (S&S Scheftner)
Chemical composition	Organic matrix: Methacrylates such as UDMA (urethane dimethacrylate), TEGDMA (triethylene glycol dimethacrylate), and Bis-EMA (bisphenol A ethoxylate dimethacrylate). Inorganic fillers: Zirconium silicate (ZrSiO₄) particles Silica (SiO₂). Nano-hybrid fillers for translucency and wear resistance. Additives: Pigments for shade matching, initiators (e.g., camphorquinone), and stabilizers.	60.2% cobalt 25% chromium 6.2% tungsten 4.8% molybdenum 2.9% gallium < 1% silicon and manganese	65% cobalt 28% chromium 9.5% tungsten 5% molybdenum 1% silicon < 1% traces (carbon, iron, manganese, and nickel)	65% cobalt 28% chromium 9.5% tungsten 5% molybdenum 1% silicon < 1% traces (carbon, iron, manganese, and nickel)
ISO classification	ISO 10,477 (polymer-based crown and bridge materials)	ISO 22,674 Type 4	ISO 22,674 Type 4	ISO 22,674 Type 4
Processing method	CAD-CAM milling	Casting	CAD-CAM milling (wet and dry)	Selective laser melting (SLM/DMLS)
Lot number	YBC32X	14,073	334,970,221	0235310221

### Construction of 3D printed resin edentulous typodont

A maxillary edentulous typodont was digitized using a desktop scanner (Medit T310; Medit Corp., Seoul, South Korea), and the resulting standard tessellation language (STL) file was imported to 3D modeling software (MeshMixer 3.1.373; Autodesk Inc., San Rafael, CA) to prepare 5 implant beds, spaced 18.5 mm between the central analog and the 2 anterior analogs and 13.5 mm between the posterior analog and its preceding analog. The STL file was then exported to an LCD 3D printer (Elegoo Mars 4 Ultra; Shenzhen Elegoo Technology Co., Egypt) to 3D-print the typodont from polymethyl methacrylate resin (MODEL; Proshape Digital Solutions, Istanbul, Turkey). Digital multi-unit analogs were inserted in implant beds, and digital scan bodies (Neobiotic IS scan body; Neobiotic, Seoul, South Korea) were attached to the analogs. The typodont was re-scanned using the former desktop scanner, and the STL file was imported to CAD software (exocad DentalCAD; exocad GmbH, Darmstadt, Germany) to design the metal framework (11.6 mm height, 31 mm width, and 39.5 mm length).

### Conventional casting group fabrication

**Fig. 1:**
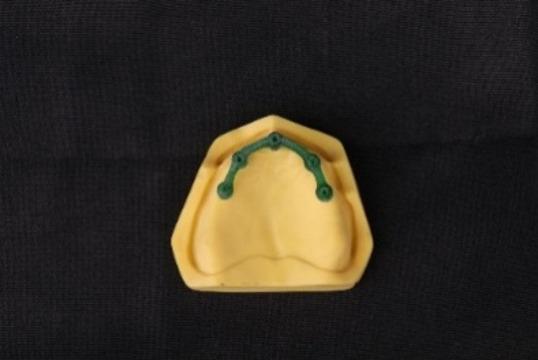
Milled wax pattern for the conventional group fits over maxillary typodont

For the conventional group, the design’s STL file was imported to a milling machine (ED5x; EMAR, Cairo, Egypt) to mill a wax pattern from incinerable acrylate polymer blanks (IPS AcrylCAD Blocks; Ivoclar Vivadent AG, Schaan, Liechtenstein). After verifying the wax pattern fit on the typodont (Fig. 1), it was sprued and invested using a phosphate-bonded material (Bellavest SH; BEGO, Bremen, Germany). Following the manufacturer’s instructions, once the investment material had set, the wax pattern underwent burnout in an oven (Miditherm 100 MP; BEGO, Bremen, Germany) at 950 °C for 60 min, and then the molten Co-Cr alloy was cast into the mold under vacuum pressure using a casting machine (Fornax T; BEGO, Bremen, Germany). After 2 h of cooling, the framework was divested and air-abraded with 250 μm aluminum oxide particles (Korox 250; BEGO, Bremen, Germany) at a pressure of 5 bars, along with glass bead abrasion at 2 bars. The sprue formers and minor surface nodules were removed with a tungsten carbide bur (Tungsten carbide bur; Edenta AG, AU, Switzerland).

### Milled group fabrication

For the milled group, the design’s STL file was imported to a 5-axis computer numerically controlled (CNC) milling machine (ED5x; EMAR, Cairo, Egypt). Each framework was wet milled from 18 mm Co-Cr blanks (Scheftner Starbond Easy disc; Schaefer GmbH, Waldshut-Tiengen, Germany), using a water-soluble synthetic coolant at a concentration of 6–8%. A total of 7 supporting arms were generated using the Millbox CAM (CIMsystem, Milano, Italy) software program, 4 on the external and 3 on the internal surfaces of each framework. Material removal followed a staged subtractive protocol using a sequence of carbide burs (Ball D3.0L10, Ball D2.0L16, Ball D1.5L12, Flat D2.0L5, Ball D1.0L10, Ball D3.0L10, Flat D1.0L14, and Flat D1.0L15) to balance sufficient bulk reduction with fine surface detailing. Milling was conducted under continuous coolant flow, with spindle speed set between 10,000 and 30,000 RPM and cutting speeds ranging from 30 to 50 mm/min along the Z axis, with higher parameters applied during roughing and reduced settings during finishing. The machine’s rapid traverse rate reached up to 4,000 mm/min. Upon completion of the 7-hour milling process, the framework was separated from the blank with a tapered tungsten carbide laboratory bur (H257EF; Komet, Lemgo, Germany), followed by conventional finishing and polishing.

### 3D printed group fabrication

For the 3D-printed group, the modified STL file was imported to the SLM 3D printing machine (VM 120; VULCANTECH GmbH, Berlin, Germany). Frameworks were 3D-printed from Co-Cr powder (Starbond Easy powder 30; Schenfer GmbH, Waldshut-Tiengen, Germany) with a particle size ranging from + 10 to -30 μm. The machine’s reservoir was filled with Co-Cr powder. A layer-wise manufacturing approach was adopted, incorporating a stripe scanning strategy with a 67º rotation between successive layers to enhance structural uniformity. The printing parameters were set to a 25 μm layer thickness and a 30º build angle [31, 32]. A 200 W high-power fiber laser fused the powder particles at a scanning speed of 1200 mm/s with a hatch distance of 0.03 mm. Another layer of the powder particles was added, and the process continues until the entire framework was completely formed, taking nearly 1–1.5 h. The framework was separated from the supporting arms using an air turbine handpiece (T3 turbine; Dentsply Sirona, Bensheim, Germany). The framework was subjected to post-processing sintering in a vacuum atmosphere furnace (Jincheng; Shanghai, China) at 900 °C for an hour [33] and finished with sandblasting by aluminum oxide 110 μm (Korox 110; BEGO, Bremen, Germany).

### Trueness assessment

**Fig. 2:**
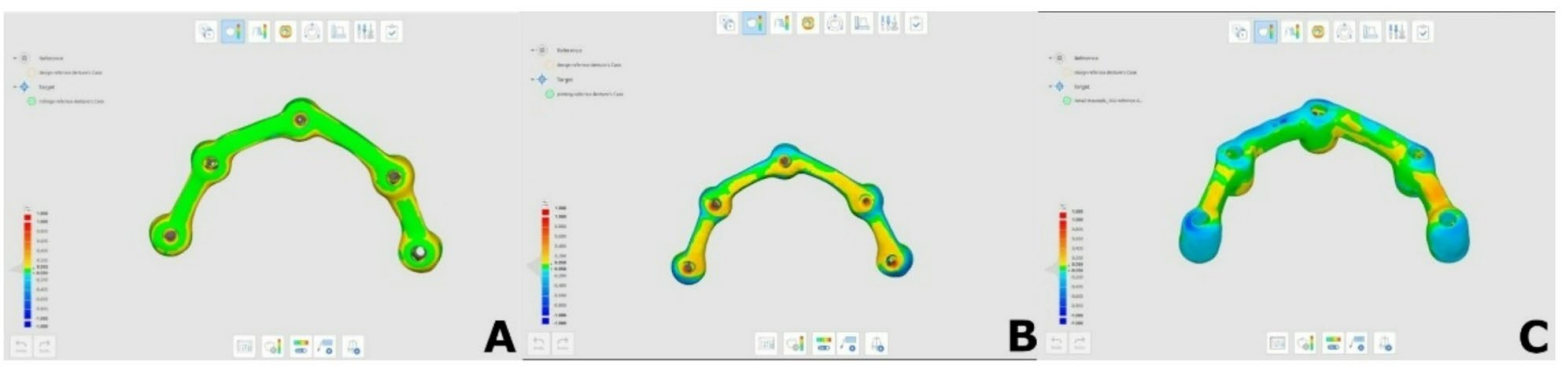
Color map for 3D deviation. **A** Milled framework. **B** Printed framework. **C** Casted framework

To assess the overall deviation (trueness) of each framework, all the frameworks were digitized using a desktop optical scanner (Medit T310; Medit Corp., Seoul, South Korea) as follows: first the occlusal aspect of the framework, then the gingival aspect. The 2 scans were merged using CAD software (Exocad DentalCAD; exocad GmbH, Darmstadt, Germany), and the resulting STL file was imported to surface matching software (Medit Link v 2.4.4; Medit Corp., Seoul, South Korea) and designated as the measured dataset, while the original design STL file served as the reference dataset. Following initial manual alignment using 3 corresponding reference points, best-fit alignment was performed. Deviations between reference and target data were visualized as a color-coded map (Fig. 2) with a deviation scale of ± 0.1 mm and a tolerance range of ± 0.05 mm. The data was expressed as root mean square (RMS) error.

### Passive fit assessment

**Fig. 3:**
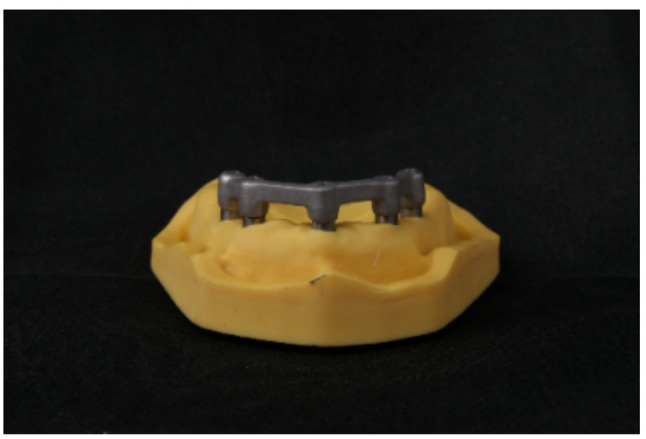
Milled framework fits over maxillary typodont

To assess passive fit, the one-screw test (Sheffield test) was performed along with a handheld digital microscope (Dino-Lite AM3111; AnMo Electronics Corporation, New Taipei City, Taiwan) mounted on a precision stand and connected to a compatible computer at 40x magnification. Each framework was first repositioned on the maxillary typodont (Fig. 3). The one-screw test is used to evaluate the passive fit of implant-supported frameworks by assessing strain-free seating under a single-screw condition and does not measure marginal fit, which is a separate geometrical parameter.

**Fig. 4:**
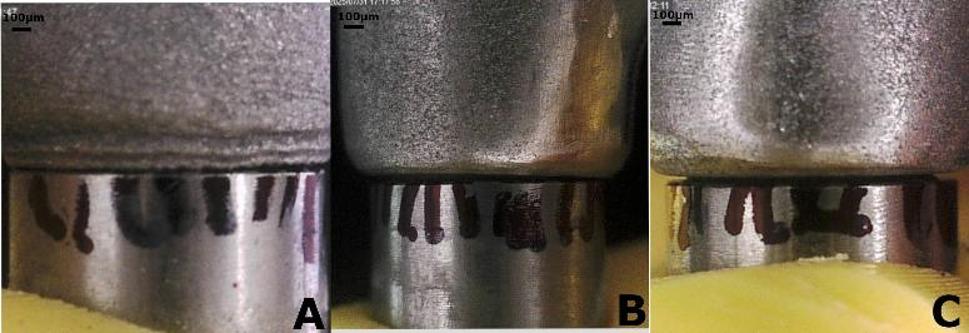
Photomicrographs of vertical marginal discrepancy under digital microscope. Original magnification ×40. Scale bar⹀ 100 μm. **A** Milled framework. **B** Printed framework. **C** Casted framework

For consistency, 15 measurement points were marked on the 1st and 5th analogs (5 buccal, 5 palatal, and 5 distal) and 10 points on the 3rd analog (5 buccal and 5 palatal). The screw of the 1st analog was tightened to a torque of 15 N.cm, and standardized digital images were obtained at the implant-framework interface of the 3rd and the 5th analogs under consistent magnification and camera position. After that, the 1st analog screw was loosened, and the 5th analog screw was tightened. Digital images at the implant-framework interface of the 1st analog were captured. Calibrated image analysis software (ImageJ 1.43u; National Institutes of Health, Bethesda, MD) was used to measure the vertical gap during passive fit at the predetermined points (Fig. 4).

### Statistical analysis

Statistical analysis was performed with a statistical software program (IBM SPSS Statistics for Windows, V23.0; IBM Corp., Armonk, NY). Data normality was confirmed by the Kolmogorov-Smirnov test. Therefore, one-way and two-way analysis of variance (ANOVA) tests were used to compare between groups, followed by Tukey’s post hoc test (α = 0.05) for pairwise comparison. The significance level was set at *P* ≤ 0.05.

A post hoc evaluation further confirmed that the selected sample size was sufficient to detect moderate-to-large effect sizes (≥ 0.65), supporting the reliability of these findings under controlled laboratory conditions.

## Results

The effect of different variables and their interactions on passive fit, as assessed using a one-screw test, is presented in Table 2. As presented in Table 3, a significant difference (*P* < 0.05) was found among the manufacturing techniques and the evaluated surfaces. The milled group showed the lowest vertical gap mean values across all surfaces, ranging from 50.50 ± 5.06 μm to 54.41 ± 3.79 μm; the 3D-printed group recorded intermediate values, ranging from 81.75 ± 3.44 μm to 83.81 ± 5.08 μm; and the conventional lost wax group exhibited the highest mean values, ranging from 125.66 ± 7.23 μm to 135.35 ± 7.17 μm.

**Table 2:** Effect of different variables and their interactions on passive fit

Source	Type III Sum of Squares	df	Mean Square	F	Sig.
Corrected Model	76114.99	8	9514.37	356.511	0.001*
Intercept	563214.22	1	563214.22	21104.077	0.001*
Group	75646.74	2	37823.37	1417.271	0.001*
Surface	61.64	2	30.82	1.155	0.322
Group * Surface	406.61	4	101.65	3.809	0.008*
Error	1681.31	63	26.69		
Total	641010.52	72			
Corrected Total	77796.30	71			

df= degree of freedom*; significant (*P* ≤ 0.05) ns; non-significant (*P* > 0.05)

R Squared = 0.978 (Adjusted R Squared = 0.976)

**Table 3:** Mean values and standard deviation of vertical gap during passive fit assessment for each manufacturing technique with Tukey post hock test results

Groups	Surface	First analog		3rd analog	5th analog	Mean value of each surface	Mean value for each group
Conventional lost wax	B	135.50 ± 14.09	139.25 ± 14.90		131.25 ± 13.47	135.35 ± 7.17 A	130.71 ± 3.94a
	D	137.75 ± 12.36	--		124.75 ± 15.86	131.25 ± 6.07 A	
	P	124.88 ± 15.36	123.75 ± 13.82		128.38 ± 12.40	125.66 ± 7.23B	
Milled group	B	50.63 ± 5.78	50.75 ± 5.60		52.00 ± 6.07	51.13 ± 2.84D	52.23 ± 2.11c
	D	51.75 ± 6.25	--		49.25 ± 6.30	50.50 ± 5.06D	
	P	57.50 ± 4.66	53.88 ± 6.33		51.88 ± 6.73	54.41 ± 3.79D	
3D-printed group	B	82.25 ± 6.07	79.63 ± 7.78		84.50 ± 6.76	82.14 ± 3.74 C	82.41 ± 2.10b
	D	85.88 ± 5.69	--		81.75 ± 6.80	83.81 ± 5.08 C	
	P	84.13 ± 5.87	86.88 ± 6.15		74.25 ± 6.14	81.75 ± 3.44 C	

*B* Buccal surface, *D* Distal Surface, *P* Palatal surface

Different capital letters indicate significant difference at (P < 0.05) among means in the same column. Different small letters indicate significant differences at (P < 0.05) among means in the same column

In the milled group, during passive fit assessment, the palatal (P) surface showed the lowest vertical gap mean value, followed by distal (D) and buccal (B) surfaces, with all surfaces exhibiting uniformly low and statistically similar values. In contrast, the conventional group displayed significantly higher vertical gap mean values, with the B surface having the lowest and the D surface the highest values. The same pattern was also observed in the 3D-printed group, with lower overall values compared to the conventional group.

On comparing the overall trueness in Table 4, the 3D-printed group showed a significantly lower mean value compared to the conventional lost wax group, while the milled group had the lowest mean value, indicating the highest trueness, with significant differences between all groups (*P* = 0.001).

**Table 4:** Mean of overall trueness and standard deviation of each manufacturing technique with Tukey post hock test results

Mean overall trueness (µm)	Conventional lost wax	Milled group	3D-printed group	F-test	*P* -value
Mean ± SD	206.50 ± 84.02a	73.50 ± 15.50c	139.63 ± 39.31b	11.999	0.001*

Different small letters indicate significant difference at (*P* < 0.05) among means in the same row, *significant (*P* ≤ 0.05)

## Discussion

This study aimed to assess and compare the passive fit and trueness of complete-arch implant-supported Co-Cr frameworks fabricated using different manufacturing techniques. Based on the findings, both null hypotheses were rejected. Significant differences existed among the fabrication techniques with respect to passive fit and trueness. The milled group reported the highest trueness and the lowest vertical marginal gap during passive fit assessment. Trueness and passive fit are closely related aspects of prosthetic framework accuracy, as any deviation from reference design may influence the ability of the framework to achieve passive, strain-free fit at the implant-prosthesis interface. This is more critical in complete-arch implant-supported prostheses, where distortion can accumulate across multiple implants’ connections and affect clinical seating. Although improved trueness is generally associated with improved passive fit, the relationship is not always direct, as the pattern of distortion may alter how discrepancies are expressed clinically. Considering both parameters can provide a more meaningful understanding of framework accuracy by linking manufacturing deviations to their clinical implications.

Cobalt-chromium alloys have been widely used due to their favorable mechanical properties and cost-effectiveness, providing acceptable marginal accuracy and long-term clinical performance [6, 16]. However, the fabrication technique remains a critical factor influencing the framework adaptation and accuracy [11].

Regarding the passive fit, the observation of this study aligns with previous reports indicating that absolute passive fit remains clinically unattainable, irrespective of the fabrication technique employed [5, 24–26]. In this study, the milling group exhibited the lowest vertical gap during passive fit evaluation, followed by the 3D printing group, while the conventional lost wax method showed significantly greater mean values. Although the differences may be statistically significant, the discrepancies among all the tested groups remained within the clinically acceptable range ]10–150 μm[ [6, 17].

These findings are consistent with Singh et al. [23], who used a one-screw test and stereomicroscope to record the passive fit of complete-arch Co-Cr frameworks on five implants. They reported that the milled group exhibited the lowest vertical gaps, followed by additive manufacturing and the conventional casting groups. In addition, El Naggar AO [28], using the one-screw test, reported that digitally fabricated milled complete-arch frameworks demonstrated improved passive fit compared with conventionally cast frameworks, further supporting the advantage of digital workflows. Other investigations, including those by AlRasheed and ALWazaan [6], Mohamed et al. [11], and the systematic review by Shah et al. [8], have compared the same fabrication techniques; however, they assessed marginal adaptation or 3D deviations, using coordinate measuring machines or microscope gap analysis under full screw tightening. Although these studies reflect marginal adaptation or geometrical accuracy rather than passive fit, their findings demonstrated a similar ranking of fabrication techniques as observed in the present study and that reported by Singh et al., with milling showing improved accuracy, followed by additive manufacturing and conventional casting. Investigations have attributed the higher adaptation in the milling group to the use of homogenous pre-sintered Co-Cr blanks with consistent microstructure and mechanical properties, reducing internal stresses, porosities, and distortions seen in casting and additive manufacturing [22, 34]. Additionally, CNC-controlled milling guided by CAD and multi-axis tool paths enhance the dimensional precision and minimize operator-dependent variability, leading to improved overall accuracy of the framework [23].

In contrast, SLM generates frameworks layer by layer, which can cause design segmentation errors and may introduce greater variance [12, 13]. As reported by literature, large SLM Co-Cr frameworks exhibit small but measurable dimensional deviations compared to short-span frameworks; this may be attributed to framework geometry, thermal accumulation and shrinkage, as well as the downstream process [11]. This also could explain the differences between the findings of this study and those conducted by Abu Ghofa and Önöral [27] and Presotto et al. [30], in which 3-unit Co-Cr frameworks were fabricated using conventional casting, milling, and SLM. In their studies, the SLM group had a superior fit compared with the milled and conventional cast groups.

To overcome these drawbacks related to SLM 3D-printed large frameworks, the thickness of the print layer was adjusted to 25 μm and the printing angle was set to 30°. The thickness of the print layer influences the fit and mechanical properties; thinner layers (20–50 μm) have been shown to improve adaptation by promoting better melting, reducing porosity, and refining microstructure through controlled heat input and cooling cycles [12, 13]. Similarly, the build orientation angle significantly impacts the fit and mechanical behavior, as it affects the way the layers are deposited, influencing layer bonding, residual stresses, and surface finish. Studies demonstrate that orientations around 30° to 45° often result in superior marginal and internal fit by minimizing stair-stepping effects and reducing post-processing requirements, while steeper angles such as 90° can increase discrepancies and stress accumulation [31, 32]. Moreover, post-processing sintering at 900 °C helped relieve residual stress and improve dimensional stability [33].

The conventional lost wax group exhibited the highest mean value of vertical gap during passive fit assessment. Although a milled wax pattern was employed to minimize operator variability and potentially improve adaptation compared with manually fabricated patterns [4, 6, 14, 22], the multiple manual steps involved, e.g., wax burnout, investing, casting, and finishing, remain prone to errors, which contributed to the greater discrepancies observed in this group [6, 11, 22].

The Sheffield one-screw test is a widely accepted clinical and laboratory method for assessing the passive fit of implant-supported frameworks, as it assesses framework seating without inducing strain [18]. A framework with a true passive fit should be fully seated without any visible gaps when secured by one screw; any observable misfit suggests fabrication inaccuracies that could lead to clinical complications [19]. In this study, the Sheffield test was combined with a high-resolution digital microscope. This microscope offers 40× to 500× magnification, allowing for precise, nondestructive, objective measurements of the vertical gap widths— which may not be apparent to the naked eye—at standardized points using image analysis software [19, 24]. This combined approach provides a comprehensive evaluation, as the Sheffield test simulates clinical seating dynamics, while the digital microscope precisely measures the vertical gap at the micrometer scale, which strengthens the reliability of passive fit evaluation and offers a clinically relevant interpretation of framework accuracy [25, 28, 35].

Assessment of the overall trueness further supported these observations, with milled frameworks exhibiting the lowest mean deviation from the reference, followed by 3D printed and conventional groups. This aligns with the expectation that digital workflows produce restorations that more closely replicate the original design. Although a significant difference was reported between the milled and SLM 3D-printed ones, both digital manufacturing techniques produced Co-Cr complete-arch implant-supported frameworks with greater trueness compared with the conventional lost wax casting technique.

The current literature on Co-Cr complete-arch implant-supported frameworks provides limited data on trueness as a defined outcome. Most investigations emphasize marginal or internal adaptation. Revilla Leon M et al. [36] and Ciocca et al. [37] have assessed 3D deviation from a reference geometry using coordinate measuring techniques, which may be interpreted as an assessment of trueness, even when not explicitly aligned with ISO 5725 terminology. Revilla Leon et al. [36] compared milled and additively manufactured Co-Cr complete-arch implant-supported frameworks and reported no significant difference between the groups, except along the X-axis, where the milled group showed lower deviation. Ciocca et al. [37] compared the milling technique with the SLM-milling hybrid technique. This hybrid workflow differs from the techniques evaluated in the present study, which limits direct comparison. Other studies have evaluated the trueness of Co-Cr frameworks fabricated using conventional casting, milling, and additive manufacturing; however, these investigations focused on removable partial denture frameworks. Heiba IM et al. [38] compared milled and additively manufactured Kennedy class I maxillary frameworks using surface matching analysis and reported higher trueness in the milled group. Sasithornvechakul C et al. [39] compared additively manufactured frameworks with conventional casting; the additive technique showed improved trueness with variations influenced by the major connector design. Similarly, Hamed HA et al. [40] reported that the frameworks fabricated using selective laser sintering exhibited greater trueness than those produced from 3D-printed wax patterns followed by casting, irrespective of the palatal vault depth. Although these studies focused on removable partial denture frameworks, their findings greatly support the influence of the manufacturing technique on framework trueness observed in the present study.

### Limitations

This study was limited by its laboratory settings, so the results may not fully reflect real-life clinical conditions. The measurement methods used, while reliable for measuring the vertical gap during the passive fit assessment and overall deviations, may not be sensitive enough to detect internal misfits or discrepancies beyond the marginal areas. In addition, the study evaluated a limited range of framework designs and implant configurations, which may limit the generalizability of the findings. Also, precision was not assessed; therefore, the reproducibility of the evaluated manufacturing techniques could not be determined.

## Conclusion

Within the limitations of this laboratory study, the following was concluded:

1. The milled Co-Cr complete-arch implant-supported frameworks demonstrated a lower vertical gap during passive fit assessment and higher trueness compared with the SLM 3D-printed and conventionally cast frameworks.
2. Although a significant difference was reported between the milled and SLM 3D-printed groups, both digital manufacturing techniques produced Co-Cr complete-arch implant frameworks with greater trueness and more favorable passive fit compared with the conventional lost wax casting technique.

### Future recommendations

Digital manufacturing techniques, such as milling and 3D printing, should be considered for the fabrication of cobalt-chromium complete-arch implant-supported prostheses due to their ability to produce frameworks with high trueness and fit. Future research should explore different framework designs, along with the variation in implant numbers and positions, as well as digital workflow parameters. In addition, further studies should focus on in vivo evaluation to validate these findings under functional conditions. Randomized clinical trials would allow the assessment of clinically relevant outcomes such as peri-implant tissue response, prosthetic complications, and patient-reported outcomes under functional loading.

## Data Availability

Available upon request from the corresponding author.

## Abbreviations

CO-CR: Cobalt Chromium
STL: Standard Tessellation Language
CAD-CAM: Computer-aided design and computer-aided manufacturing
SLM: Selective Laser Melting
CNC: Computer Numerical Control
RMS: Root Mean Square
P: Palatal surface
D: Distal surface
B: Buccal surface
